# Culture and mental health in Nepal: an interdisciplinary scoping review

**DOI:** 10.1017/gmh.2018.27

**Published:** 2018-11-05

**Authors:** L. E. Chase, R. P. Sapkota, D. Crafa, L. J. Kirmayer

**Affiliations:** 1 McGill University - Global Mental Health Program, Montreal, Quebec, Canada; 2 Division of Social and Transcultural Psychiatry, McGill University, Montreal, Canada; 3 Integrated Program in Neuroscience, McGill University, Montreal, Canada

**Keywords:** Culture, global mental health, interdisciplinary, Nepal, other, review

## Abstract

Efforts to address global mental health disparities have given new urgency to longstanding debates on the relevance of cultural variations in the experience and expression of distress for the design and delivery of effective services. This scoping review examines available information on culture and mental health in Nepal, a low-income country with a four-decade history of humanitarian mental health intervention. Structured searches were performed using PsycINFO, Web of Science, Medline, and Proquest Dissertation for relevant book chapters, doctoral theses, and journal articles published up to May 2017. A total of 38 publications met inclusion criteria (nine published since 2015). Publications represented a range of disciplines, including anthropology, sociology, cultural psychiatry, and psychology and explored culture in relation to mental health in four broad areas: (1) cultural determinants of mental illness; (2) beliefs and values that shape illness experience, including symptom experience and expression and help-seeking; (3) cultural knowledge of mental health and healing practices; and (4) culturally informed mental health research and service design. The review identified divergent approaches to understanding and addressing mental health problems. Results can inform the development of mental health systems and services in Nepal as well as international efforts to integrate attention to culture in global mental health.

## Introduction

While mental health problems are increasingly recognized as a global health priority, debate continues over the relevance of cultural variation for the application of psychiatric diagnoses and treatments (Chisholm *et al*. 2007; Collins *et al*. 2011; Patel *et al*. 2011; Whitley, 2015). This debate has taken on renewed importance with recent calls by global mental health advocates for rapid scale-up of mental health services in low- and middle-income countries (Patel *et al*. 2011, 2016; Bhugra *et al*. 2017). Cultural and contextual factors are now understood to influence every aspect of mental health and illness (Alarcón *et al*. 2009; Kirmayer, 2013; Napier *et al*. 2014). Diagnostic and treatment guidelines and frameworks for mental health systems recognize cultural variation in the manifestation of distress and disorders and call for culturally appropriate interventions (Psychosocial Working Group, 2003; Inter-Agency Standing Committee (IASC), 2007; American Psychiatric Association, 2013; World Health Organization, 2013*b*; Khenti *et al*. 2016).

Yet critics have pointed to a gap between the rhetoric of global mental health, which recognizes the importance of social and cultural context, and practice, in which local knowledge is often overlooked in favor of a generic biomedical psychiatric or psychosocial approach (Watters, 2010; Campbell & Burgess, 2012; Summerfield, 2012; Clark, 2014; Fernando, 2014; Mills, 2014; Bracken *et al*. 2016). There is continued concern over whether methods of research, diagnosis, and treatment that have been developed primarily in urban, high-income, and industrialized settings will meet the needs of populations in which the greatest mental health disparities are found. Failure to adequately address social and cultural contexts may limit the effectiveness of interventions and have other negative impacts, including promoting the medicalization of social suffering and undermining indigenous knowledge and support systems (Desjarlais *et al*. 1995; Argenti-Pillen, 2003; Clark, 2014; Whitley, 2015). The literatures of medical anthropology and sociology contain rich descriptions of many settings where global mental health is active; however, the generalizability of these accounts and their relevance to current mental health issues are not always clear to practitioners (Greene *et al*. 2017).

Nepal offers a useful case study in this discussion because it has seen many decades of social science research and there is a current need for information to guide ongoing efforts to scale up mental health services. A small landlocked country of about 29 million people with the third lowest human development rating in South Asia (United Nations Development Programme, 2016), Nepal has about 110 psychiatrists, 15 clinical psychologists, and 400–500 paraprofessional psychosocial workers (Luitel *et al*. 2015; Sherchan *et al*. 2017). Government investment in mental health in Nepal has historically been very limited (around 0.7% of the health budget), with more than half of available services provided by non-governmental organizations (NGOs) [World Health Organization (WHO), 2011; Inter-Agency Standing Committee (IASC) Reference Group for Mental Health and Psychosocial Support in Emergency Settings, 2015].

The development of formal mental health services in Nepal has been led by an array of local and international humanitarian actors over the past four decades. The WHO began mental health work in Nepal in 1980 and the United Mission to Nepal launched the first community mental health services in 1984 (Acland, 2002). The first mental health NGOs were established in the 1990s to treat those affected by the ongoing Maoist insurgency and the mass influx of refugees from Bhutan (Jordans & Sharma, 2004; Tol *et al.* 2005; Jordans *et al*. 2007; Center for Victims of Torture Nepal, 2011). With the end of the civil conflict in 2006, mental health NGOs and advocates shifted their focus to strengthening the mental health system (Upadhaya *et al*. 2014). Nepal became an implementation site for several high-profile global mental health projects (Hanlon *et al*. 2014; Mendenhall *et al*. 2014; Kohrt *et al*. 2015; Jordans *et al*. 2016). In 2015, Nepal was struck by a major earthquake, inspiring a proliferation of mental health and psychosocial projects (Seale-Feldman & Upadhaya, 2015). The financial resources and political will elicited by the disaster contributed to advancing national global mental health agendas (Chase *et al*. 2018).

While Nepal has been a popular site for research on social, cultural, and ritual aspects of healing, few attempts have been made to consolidate this body of work or explore its relevance to ongoing health development initiatives. A desk review published shortly after the 2015 earthquake drew attention to existing literature on cultural aspects of mental health in Nepal (IASC Reference Group for Mental Health and Psychosocial Support in Emergency Settings, 2015). However, this review was not conducted with the methodological rigor of a scholarly report and did not capture publications produced during the period of heightened interest and investment in mental health following the disaster. The present scoping review, completed 2 years after the earthquake, takes stock of the current state of scholarship on culture and mental health in Nepal, including relevant literature from across the health and social sciences.

## Methods

We employed a scoping review methodology (Arksey & O'Malley, 2005) with the guiding research question: ‘What knowledge exists on the relationship between culture and mental health in Nepal?’ For the purposes of this review, mental health was interpreted as encompassing mental health/wellbeing and mental illness/disorder, where ‘psychiatric’, ‘psychological’, and ‘psychosocial’ were acceptable replacements for ‘mental’. Culture was interpreted as ‘values, beliefs, knowledge, norms, and practices and the notion that these are shared among a specific set of people’ (Hruschka & Hadley, 2008, p. 947). Publications addressing social and structural issues that exist in many societies (e.g. gender inequality and mental health stigma) that were not explicitly linked to Nepali culture in some way were excluded. Publications addressing culture among ethnic minority groups in Nepal and ethnically Nepali Bhutanese refugees were included, while those focused exclusively on populations living outside Nepal were excluded.

Searches were carried out in collaboration with a medical librarian in PsycINFO, Web of Science, Medline, and Proquest Dissertation. Terms used to study mental health and illness in both social science (e.g. ‘healing’) and clinical (e.g. ‘treatment’) fields were included, as were transliterated Nepali words commonly referenced in the mental health literature [e.g. *sato*, meaning ‘spirit or soul’ as used in the Nepali idiom ‘soul loss’ (see Kohrt & Hruschka, 2010); *chhopne*, literally ‘to catch, to get hold of, and to cover by someone or something’, used to describe experiences of dissociation or possession (Sapkota *et al*. 2014, p. 645); NB: definitions may vary according to the context and ethnic/linguistic group]. The following search terms were used in all databases: (Mental* OR Madness OR Psycholog* OR Distress* OR Idioms* OR Caus* OR Cultur* OR Belief* OR help seeking OR Healing OR Somatic* OR Possession OR Soul* OR Spirit* OR Sato* OR Rog* OR Dokh OR Psychosocial* OR Counsel* OR Witch* OR Ritual OR Chhopne OR Ethno* OR Festival OR Treatment) AND Nepal*. Texts published up to 22 May 2017, when the searches were completed, were included in this review.

Results from the searches were screened according to the following inclusion criteria: (1) English or Nepali; (2) peer-reviewed journal article, book/book chapter, or doctoral thesis; and (3) substantial original discussion of culture in relation to mental health in Nepal. Initial screening of titles and abstracts was carried out by bilingual (English/Nepali) team members with graduate training in transcultural psychiatry. All full texts of publications appearing to meet criteria were then screened by two team members. When there was disagreement among reviewers on whether a publication met inclusion criteria, additional coauthors reviewed the full text and consensus was reached through discussion. Additional items were identified by screening the reference lists of all included texts, the aforementioned desk review (IASC Reference Group for Mental Health and Psychosocial Support in Emergency Settings, 2015), and a bibliography of psychological research in Nepal (Maharjan, 2012).

Texts meeting inclusion criteria were divided among team members for ‘charting’ (Arksey & O'Malley, 2005): key information was extracted using a form covering methods, focus, and key findings; reviewers also indicated whether the text reflected an applied orientation, defined as including discussion of how findings could inform or improve mental health services for culturally Nepali populations. Finally, texts were collated and summarized (Arksey & O'Malley, 2005) by the first author with input from other team members.

## Results

**Fig. 1.**
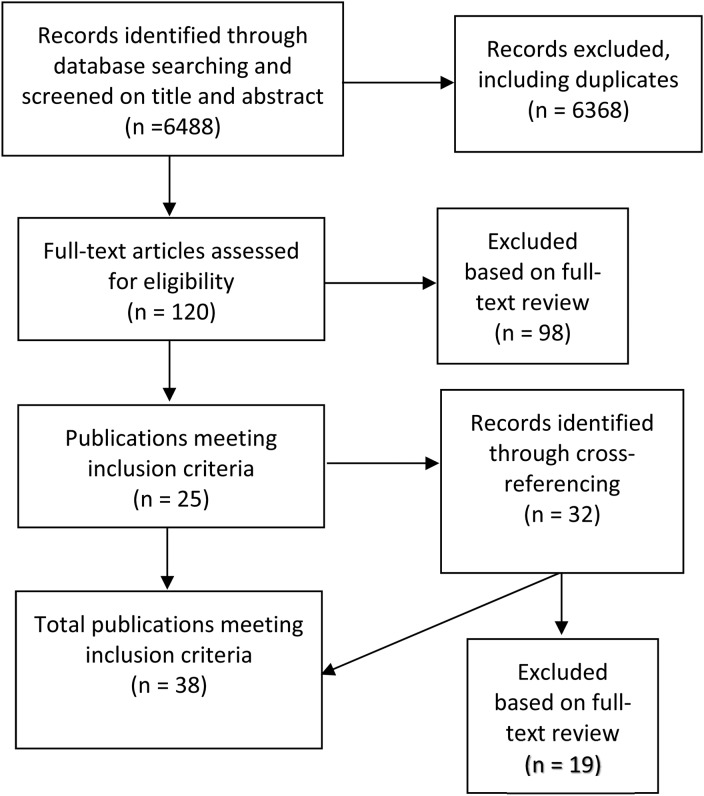
Search strategy.

The search yielded 6488 results, of which 38 met inclusion criteria (see Fig. 1 and Table 1 for an overview of the search process and included texts). To facilitate practical use of this review, we have collated publications into four thematic categories.

**Table 1.** Overview of texts included in the review

Reference	Type	Population	Methods	Relevant topics covered	Applied orientation
Adhikari *et al*. (2015)	Article	Children (*n* = 24), parents (*n* = 48), schoolteachers (*n* = 8), and key informants (*n* = 22)	Free list interviews and key informant interviews	Interpretations of and responses to child behavioral problems	Yes
Boehnke *et al*. (1998)	Article	Undergraduate students in Nepal (*n* = 530) and two other countries	Schwartz Value Survey, Goldenring-Doctor Scale of Existential Worries, and various measures of mental health	Influence of cultural values on individual worries and mental health outcomes	Yes
Böker (1992)	Article	Psychiatric patients at a government mental hospital and their relatives (*n* = 110)	Semi-structured/narrative interviews	Concepts of and attitudes toward mental illness, help-seeking pathways, causal attributions	Yes
Bragin *et al*. (2014)	Article	Women aged 18–25 and key informants in Nepal (*n* = 437) and two other countries	Qualitative phenomenological: stepwise ethnographic exploration and aspects of the participatory ranking method (focus groups and interviews)	Cultural concepts of psychosocial wellbeing	Yes
Burkey *et al*. (2016*a*)	Article	Parents, teachers, and peers (*n* = 30); children (*n* = 60)	Free lists, interviews with parents and teachers, pilot testing of Disruptive Behavior international Scale-Nepal version (DBIS-N)	Cultural concepts of child behavior problems and overlap with Western diagnostic criteria	Yes
Burkey *et al*. (2016*b*)	Article	Parents, teachers, and community leaders familiar with child-rearing (*n* = 40) and children (*n* = 9)	In-depth interviews and focus groups, pile sort interviews, and direct observations	Cultural concepts of child behavior problems and appropriate responses	Yes
Chase & Bhattarai (2013)	Article	Bhutanese refugees in Nepal and the USA (*n* = 62)	Ethnographic (semi-structured interviews and participant observation)	Cultural concepts of wellbeing and ethnopsychology of resilience	Yes
Clarke *et al*. (2014)	Article	Distressed mothers, traditional healers, and community members (*n* = 22)	Semi-structured interviews, grounded theory analysis	Cultural concepts of distress, explanatory models, help-seeking pathways	Yes
Evers *et al*. (2016)	Article	Tharu ethnicity children, parents, and community members exposed to civil conflict	Focus groups, individual interviews, inventory of children's daily activities and walkabouts	Cultural concepts of trauma, importance of living and dead relations	Yes
Furr (2004)	Article	Teachers in Nepal (*n* = 276)	Self-developed instrument gauging ‘Western orientation’ and tendency to medicalize	Concepts of mental illness and medicalization	No
Furr (2005)	Article	Teachers in Nepal (*n* = 276)	Self-developed instrument gauging ‘Western orientation’ and Costello–Comprey Depression and Anxiety Scale	Relationship between cultural values and mental health	No
Harper (2014)	Book chapter	Health professionals, traditional healers, and patients	Ethnography (interviews and participant observation)	Traditional healing methods, illness categories, somatization, causal attributions	No
Heys *et al*. (2017)	Article	Parents of autistic and non-autistic children and health and education professionals (*n* = 106)	Focus groups and semi-structured interviews	Knowledge and awareness of autism and its impacts	Yes
Hoge *et al*. (2006)	Article	Outpatients with general anxiety disorder in Nepal (*n* = 30) and America (*n* = 23)	Beck Anxiety Inventory questionnaire	Cultural differences in presentation of anxiety	Yes
Jack *et al*. (2010)	Book chapter	Masters and undergraduate students (*n* = 95; for instrument adaptation); male and female clients of outpatient psychiatric clinics (*n* = 96)	Instrument adaptation through translation monitoring process and testing (van Ommeren *et al*. 1999), semi-structured interviews, focus groups, Composite International Diagnostic Interview (CIDI) and Silencing the Self Scale based self-reports	Relationship between cultural gender norms and depression	No
Jolly (1999)	Article	One Nepali soldier in British army	Case study	Illness concepts, traditional healing	No
Jordans *et al*. (2003)	Article	N/A	Reflection on experience adapting and implementing psychosocial counsellor training	Cultural adaptations for counselling in Nepal, psychosocial problems specific to cultural context	Yes
Kaplan (1999)	Doctoral thesis	Adults (*n* = 390)	Structured interview including Nepali Psychiatric Symptom Checklist and questions about causes, effects, and treatments of the symptoms	Beliefs about meaning of psychiatric symptomology and appropriate treatments	Yes
Kim *et al*. (2017)	Article	Widows and key informants (*n* = 37 for interviews; *n* = 20 for focus groups)	Semi-structured interviews and focus group discussions using the Explanatory Model Interview Catalogue	Cultural concepts of grief and grief-related pathology	Yes
Kohrt & Bourey (2016)	Article	Female child soldiers in Nepal (*n* = 13)	Structured vignette interviews	Influence of cultural context on comorbid mental health and reproductive health problems	Yes
Kohrt & Harper (2008)	Article	Health professionals (including traditional healers), clients, and lay community members	Literature review and ethnographic research including interviews and participant observation	Concepts of self and mind–body connection, help-seeking pathways, stigma	Yes
Kohrt & Hruschka (2010)	Article	Lay community members and professionals in psychosocial organizations in Kathmandu	Semi-structured interviews, survey (comprising free lists and an emotion questionnaire), comparison tasks, and observant participation	Concepts of trauma and vulnerability to trauma, idioms of distress	Yes
Kohrt & Maharjan (2009)	Article	Key informants from 10 districts of Nepal (*n* = 21)	Key informant interviews	Ethnopsychology of child development and violence	Yes
Kohrt *et al*. (2012)	Article	Bhutanese refugees	Theoretical discussion	Culturally adapted psychotherapeutic interventions	Yes
Kohrt *et al*. (2005)	Article	Adults (*n* = 316; subgroup of 65 participants with *jhum-jhum*)	Standard interview process including questions about life history, depression (BDI), anxiety (BAI), and stressful life events (SLERS); ethnographic history; and medical exam	Cultural differences in somatization: relationship of *jhum-jhum* (common somatic complaint) with depression	Yes
Kohrt *et al*. (2009)	Article	Adults (*n* = 307, high and low castes)	Ethnography, Beck Depression and Beck Anxiety Inventories	Relationship between caste and mental health	Yes
Kohrt *et al*. (2011)	Article	Children (*n* = 64 for focus groups during transcultural translation process; *n* = 162 for validation)	Transcultural translation and validation of Depression Self-Rating Scale (DSRS) and Child PTSD Symptom Scale (CPSS), validated using Kiddie-Schedule for Affective Disorders and Schizophrenia and Global Assessment of Psychosocial Disability	Transcultural translation and adaptation of instruments, concepts of depression and trauma	Yes
Kohrt *et al*. (2016)	Article	Representative adult sample for focus groups during transcultural translation process (*n* = 38); primary care patients for validation (*n* = 125)	Transcultural translation and administration of Patient Health Questionnaire (PHQ-9) and two screening items based on idioms of distress; CIDI used to validate	Idioms of distress, transcultural translation and adaptation of instruments	Yes
Kohrt (2009)	Article	Adults (*n* = 316, high and low castes)	Historical discourse analysis and General Health Questionnaire	Relationship between caste and mental health	Yes
Kohrt (2015)	Book chapter	Children, community members, and key informants (*n* = 152 for interviews; *n* = 24 for case studies, *n* = 142 for survey)	Narrative focus group discussions (25 groups), key informant interviews, case studies and quantitative survey of child soldiers	Relationship between traditional rituals and psychosocial wellbeing	Yes
Pach III (1998)	Book chapter	Villagers described as being *baulāhā* (mad) and other community members	Surveys, ethnographic interviews, and observation	Cultural concepts and social implications of madness	No
Peters (1978)	Article	Tamang ethnic group in Nepal	Interviews and observation	Traditional healing system (shamanism) and parallels with psychotherapy	No
Peters (1981)	Book	Tamang ethnic group in Nepal	Interviews and observation	Traditional healing system (shamanism) and parallels with psychotherapy	No
Sapkota *et al*. (2014)	Article	Possessed and non-possessed community members, their family members, and traditional healers	Pilot study, case–control study, focus groups	Cultural context and psychosocial factors associated with spirit possession	Yes
Sharma & van Ommeren (1998)	Article	Tortured Bhutanese refugees in Nepal (*n* = 10 for narrative analysis, *n* = 25 for case notes)	Narrative analysis, analysis of case notes, focus groups	Idioms of distress	Yes
Skultans (1988)	Article	Patients of a tantric healer (*n* = 137) including 69 with mental illness and patients of an outpatient clinic of a mental hospital (*n* = 69)	Interview	Indigenous healing (shamanism), causal attributions	No
Soubrouillard (1995)	Doctoral thesis	Shamans/faith healers (*n* = 5)	In-depth interviews with healers	Indigenous healing and mental illness, causal attributions	No
Tol *et al*. (2005)	Article	Clients in psychosocial counselling	Case studies	Counselling in Nepali cultural context	Yes

### (1) Cultural determinants of mental health problems

Six publications addressed how culture influences the etiology of mental distress and disorder, in particular by shaping patterns of social organization and inequality. Kohrt *et al*. (2009) examined the correlation between low caste and rates of depression and anxiety, identifying poverty, lack of social support, and stressful life events as mediators. In a related article, Kohrt (2009) interpreted associations between low caste and psychological morbidity though a policy analysis, highlighting the role of restrictions in social life and access to resources that affect low caste groups in the Nepali context. A book chapter by Jack *et al*. (2010) described how traditional Hindu Brahmanical models of the ‘good woman’ may lead to self-silencing and consequently contribute to depression among women in Nepal. Similarly, Kohrt & Bourey (2016) explored how cultural norms related to gender (e.g. perceived lack of autonomy, lack of social support for women leaving a marriage) contribute to the risk for comorbid maternal mental illness by influencing exposure to intimate partner violence.

Two articles considered how cultural values contribute to the development of mental health problems. Furr's (2005) sociological study found that ‘Western orientation’, as measured through a self-developed questionnaire assessing attitudes toward gender and caste norms and language/media preferences, was associated with lower depression scores. In a comparative study, Boehnke *et al*. (1998) found that Asian samples (Nepali and Fijian) valued tradition, conformity, and power more highly than Europeans (German) and had more microsocial (personal) than macrosocial (e.g. national or environmental) worries. In Nepal, microsocial worries were negatively related to mental wellbeing, but there was no direct relationship between cultural values and wellbeing.

### (2) Culture and mental illness experience

A second set of 14 publications framed culture as a set of beliefs and values that shape mental illness experience. Several focused specifically on the ways that mental health problems manifest or are expressed in Nepali cultural contexts. Sharma & van Ommeren (1998) identified salient ‘idioms of distress’ (Nichter, 1981) among Bhutanese refugee torture survivors, including emotion-related idioms (e.g. *dukha lāgyo* or sadness) and somatic idioms (e.g. headache, dizziness); many attributed their suffering to bad deeds committed in previous lives (*karmako phal*). Hoge *et al*. (2006) found that Nepalis with generalized anxiety disorder showed more somatic symptoms and fewer psychological symptoms compared with Americans and offered possible explanations related to cultural differences in stigma, mind–body distinctions, introspection, and acceptable means of expressing distress. By contrast, Kohrt *et al*.’s (2005) work on *jhum-jhum* (a common somatic complaint in Nepal involving numbness or tingling) among depressed patients found that once the local burden of physical illness had been accounted for, rates of somatization in Nepal were comparable to those in Western settings.

Five studies explored how culture mediates the interpretation of particular symptoms and behaviors as deviant or pathological. Furr (2004) found that teachers with a more ‘Western’ orientation according to the aforementioned measure (Furr, 2005) were more likely to pathologize deviant child behavior. Adhikari *et al*. (2015) identified behaviors commonly reported as problematic among children in Nepal (e.g. addiction, negligence of studies, anger); these were mainly attributed to the social environment and intervention strategies ranged from talking to physical punishment. Burkey *et al*. (2016*b*) identified local social goals and gender norms that influenced when specific child behaviors were deemed pathological. Heys *et al*.’s (2017) study of Nepali understandings of autism identified beliefs that could interfere with help-seeking, especially attributions to poor parenting. Finally, Kim *et al*. (2017) examined which manifestations of grief among Nepali widows were locally considered pathological, finding some overlap with criteria for persistent complex bereavement disorder (e.g. prolonged duration, role/identity confusion, impaired daily functioning, mistrust).

More generally, Böker (1992) explored concepts of mental illness among mental hospital patients (likely suffering from severe/psychotic disorders) and their relatives. Most attributed the illness to spirit possession, physical problems in the body, fever, and separation or conflicts within the family. Pach III's (1998) book chapter explored Nepali villagers’ perceptions of individuals described as being *baulāhā* (mad), finding that afflicted individuals experienced social marginality that constrained their access to care and was at times more distressing than the illness itself. Kaplan's (1999) doctoral thesis explored the meaning of psychiatric symptomatology in rural Nepal, including common supernatural attributions (e.g. witchcraft, the intervention of ghosts and spirits), as well as treatment modalities believed to address these causes. Clarke *et al*. (2014) studied Nepali mothers’ concepts of psychological distress; distress was often attributed to family- and gender-related factors and women's responses to it were shaped by a fatalistic worldview.

Finally, two publications addressed indigenous illness categories. Sapkota *et al*. (2014) examined mental health factors associated with unintentional spirit possession. They argue that rather than mapping onto a single diagnostic category, spirit possession may function as an idiom of distress facilitating expression of ‘suffering related to mental illness, socio-political violence, traumatic events, and the oppression of women’ (p. 643). Evers *et al*. (2016) used the case of ‘soul loss’ in the wake of Nepal's civil conflict to illustrate how socially and spiritually anchored conceptions of the self influence the experience of psychopathology and the course of healing.

### (3) Cultural knowledge of mental health and healing

A third set of 11 publications explored cultural knowledge on mental health and healing in Nepal. Several of these document aspects of Nepali ‘ethnopsychology’, or ‘cultural concepts of self, mind-body divisions, emotions, human nature, motivation, and personality’ (Kohrt & Maharjan, 2009, p. 115). Kohrt & Harper (2008) elaborated elements of self that are ‘central to understanding conceptions of mental health and psychological wellbeing and subsequent stigma’ (p. 468) in Nepal, including the soul, heart–mind, brain–mind, body, and social status. In a 2009 publication, Kohrt and Maharjan provided an overview of Nepali concepts of child development and the perceived effects of violence and psychological trauma. Kohrt & Hruschka (2010) explored Nepali concepts of psychological trauma and associated idioms of distress and emotion terms. They highlight how the attribution of traumatic experiences to one's actions in a past or present life (*karma*) can lead to blame and stigma, with implications for help-seeking.

Three studies explored cultural concepts of and pathways to wellbeing. Bragin *et al*. (2014) identified locally salient domains of wellbeing in three conflict-affected countries (including, specific to Nepal, having all basic needs met and freedom of movement) and describe the influence of spiritual traditions on understanding wellbeing, evident in the use of terms such as *ānanda* (transcendent bliss). Chase & Bhattarai (2013) explored resilience among Bhutanese refugees in the USA and Nepal; they present idioms of wellbeing and describe processes that promote resilience, such as daily worship (*pūjā*) and involvement in community groups. Finally, a book chapter by Kohrt (2015) describes how the practice of certain Nepali traditional rituals can promote psychosocial wellbeing, particularly during reintegration of child soldiers. For example, *Swasthāni*, a fasting ritual performed by women for the wellbeing of male relatives and atonement of sins, may lead to increased acceptance of girl soldiers.

Several publications addressed expert or esoteric cultural knowledge – that of practitioners of indigenous healing systems. Peters’ article (1978) and subsequent book (1981) on the Tamang ethnic group draws parallels between techniques used by shamans and those of Western psychotherapy, including mediation in social conflict, facilitation of catharsis, and providing a symbolic structure for understanding illness. Skultans (1988) compared the practice of a psychiatric outpatient clinic with that of a ‘tāntrik healer’ – a type of healer known for ‘sweeping away the negative forces believed to account for ill health or misfortune, and blowing on the positive and regenerative forces in their place’ (*jhar-phuk*; Dietrich, 1998, p. ix). The healer had adopted elements of psychiatric practice (e.g. speed and standardization) but offered causal attributions that were more likely to reinforce family support. Soubrouillard's doctoral thesis (1995) explored how Nepali shamans understand, assess, attribute and treat madness; diagnostic methods including divination are described in detail. Finally, Jolly (1999) described how a Nepali soldier in the British army was cured of psychiatric symptoms by visiting a traditional healer, noting parallels with the practice of mental health professionals. The study by Böker (1992) described above also discusses treatment-seeking pathways, including preferences for traditional healers.

### (4) Culturally informed mental health care

Finally, six publications explored how attention to culture can be integrated into the detection and treatment of mental health problems. With regard to detection, Kohrt *et al*. (2011) proposed six evaluation questions to guide cross-cultural validation of instruments for child mental health research. Using the examples of the Depression Self-Rating Scale (DSRS) and Child PTSD Symptom Scale (CPSS), they demonstrate how these questions can guide effective translation of instruments by trained mental health paraprofessionals and discuss adaptations made (e.g. incorporation of pictographic scales). Kohrt *et al*. (2016) adapted the PHQ-9 for use in Nepal, developed two additional questions based on local idioms of distress, and validated these among primary care patients. They determined that an algorithm involving initial screening for heart–mind problems and impaired functioning could improve the efficiency and accuracy of screening with PHQ-9. Burkey *et al*. (2016*a*) explored the overlap between local categories of problematic behavior and Western diagnostic criteria as reflected in the Disruptive Behavior international Scale-Nepal version (DBIS).

With regard to treatment, Harper's (2014) book chapter examined how United Mission to Nepal introduced psychiatric services and psychotropic drugs in Nepal, describing how diagnostic procedures were adapted to the local cultural context; for example, the label of ‘nerves disease’ (*nasā rog*) was used to make depression treatment more socially acceptable. Jordans *et al*. (2003) described cultural adaptations made by the Centre for Victims of Torture, Nepal (CVICT; see also Sharma & van Ommeren, 1998) in training psychosocial counsellors, including demonstrating respect for clients’ social status, applying indirect ways of questioning, and specialized training modules focused on stigmatization and supernatural attributions. Tol *et al.* (2005) outlined some ‘cultural challenges’ CVICT faced in establishing psychosocial counselling in Nepal, as well as adaptations made to address issues related to the therapeutic relationship, illness beliefs, locus of control, and views of the self and introspection. Finally, Kohrt *et al*. (2012) discuss possible adaptations to cognitive behavior therapy, interpersonal therapy, and dialectical behavior therapy to accommodate Nepali ethnopsychology, with the goal of improving care of Bhutanese refugees.

## Discussion

This scoping review identified a modest body of literature on culture and mental health in Nepal. Publications represented a range of disciplines and research methods. Culture was investigated variously as a contributor to mental illness, a set of beliefs and values that shape mental illness experience and help-seeking, a repository of indigenous knowledge about mental health, and a factor that could be effectively integrated in mental health research and service design. Much of the literature in this area published before 2015 was synthesized in the post-earthquake desk review (IASC Reference Group for Mental Health and Psychosocial Support in Emergency Settings, 2015). However, it is striking that nearly 25% of identified texts were published since the beginning of 2015. This may reflect growing interest in the critical role of social context in mental health and illness (Tol *et al.* 2010) as well as the increased attention to mental health issues occasioned by disasters (World Health Organization, 2013*a*).

Nearly 75% of included publications reflected an applied orientation. Many of these were published within the past 15 years by researchers affiliated with Nepali mental health NGOs. Some applied work strived to directly integrate culture into mental health research and practice with the goal of improving quality of and access to services. Other studies conducted in affiliation with these NGOs adopted a broader focus, contributing to our understanding of Nepali concepts of distress, pathology, trauma, child development, and resilience.

At the same time, we noted that applied research (with a few important exceptions such as the work on ethnopsychology) tended to be structured around concepts emerging from globalized psychiatric knowledge, with accounts of culture often reduced to one or two dimensions that shaped or interfered with conventional methods of research and practice. Only three studies were framed around indigenous illness categories (Kohrt *et al*. 2005; Sapkota *et al*. 2014; Evers *et al*. 2016). All of the included research on traditional healing was conducted prior to the year 2000, despite the fact that these healers continue to be the primary source of treatment for mental health problems in Nepal (Luitel *et al*. 2015). In some cases, narrow and essentializing conceptualizations of ‘Nepali culture’ were evident. For example, Furr (2004, 2005) considered willingness to support female political leaders to be an indication of ‘Western’ (as opposed to ‘Nepali’) cultural orientation – yet, Nepal has now elected a female president well in advance of many Western countries. Moreover, there is evidence that cultural beliefs and idioms take on new meanings when instrumentalized within mental health diagnosis and service provision, raising questions about the limits of culturally adapted interventions (Abramowitz, 2010). There remains a need for long-term ethnographic research that examines local understandings and experiences of mental health problems. In addition to improving our understanding of the contexts and consequences of global mental health interventions, studies of this nature may shed light on underlying processes of psychopathology and intervention strategies grounded in local ethnopsychologies and indigenous healing systems that can contribute to a truly global psychiatry (Chase & Sapkota, 2017).

The body of literature outlined here can and should inform mental health policy and practice in Nepal (Kirmayer & Pedersen, 2014). This review comes at a critical historical moment: Nepal's government has allocated a budget for mental health care at the district level for the first time, revised mental health policy is pending after 20 years, and the Ministry of Health has demonstrated a commitment to addressing mental health in the context of its action against non-communicable diseases (Chase *et al*. 2018; Government of Nepal, 2014). Findings may be relevant to a range of stakeholders involved in the anticipated scaling up of services. The national baseline psychiatric epidemiological study that is currently being planned may use instruments described here that have undergone a culturally informed validation process. The wealth of information identified in this review about idioms of distress, concepts of causality, and indigenous illness categories could enhance the assessment of mental health problems, and should thus inform future revisions of the Standard Treatment Protocol for mental health in primary care as well as efforts to improve and contextualize medical school curricula on mental health and mhGAP-based trainings for physicians. Clinicians working in Nepal should consider incorporating the cultural adaptations to psychological and psychosocial treatments documented above.

One possible barrier to the application of research findings is disciplinary variation in jargon and writing conventions; social scientists seeking to inform practitioners should consider publishing versions of their findings in clear accessible language (Greene *et al*. 2017). Working in collaboration with patients, clinicians, policy makers and other knowledge users may help researchers find the appropriate vocabulary to translate their findings into practical applications. The recent development of a ‘community informant detection tool’ in Nepal (Subba *et al*. 2017; published shortly after our review) offers an excellent model for collaborative, culturally informed mental health work of this nature.

Finally, it is noteworthy that only three included publications had a Nepali first author. The small number of Nepali scholars who have published in this area has implications for the peer review process, as relying mainly on non-Nepali reviewers could result in the dissemination of limited or misleading interpretations of local terms and perspectives. Findings of this review thus lend support to calls for greater representation of scholars from low-income countries in the global mental health literature (Kohrt *et al*. 2014).

## Limitations

This scoping review has several limitations. Nepali language search terms could have been transliterated in multiple ways and we did not include search terms in Devanagari script. Following Arskey & O'Malley (2005), this study did not assess the quality of included texts; caution is therefore needed in applying findings. In addition to considering information summarized in Table 1, readers should refer to the full texts of publications. Our stringent inclusion criteria may have led to the exclusion of some relevant literature. Some potentially relevant work on emotions, coping, and self-hood that did not make an explicit link to mental health and illness was excluded (e.g. Mchugh, 1989; Cole & Tamang, 1998; Cole *et al*. 2002; Chase *et al*. 2013). Some instrument validation studies that did not meet the criterion of ‘substantial original discussion’ of culture and mental health did make references to the culture and context and may be relevant for researchers planning to use these scales in Nepal (e.g. Kohrt *et al*. 2002, 2003; Haroz *et al*. 2017; Sochos & Lokshum, 2017; see Chen *et al*. 2013 for more on psychiatric scales used in Nepal). We recommend that all future studies reporting successful cross-cultural validation of instruments describe the translation process and adaptations made in detail. Finally, we note the exclusion of a rich body of ethnographic literature that explores suffering and healing in local Nepali terms, without employing the language of mental health and illness (e.g. Hitchcock, 1967; Hitchcock & Jones, 1976; Stone, 1976; Desjarlais, 1989, 1992; Maskarinec, 1992; Subba, 2007). More work is needed on ways to integrate this rich body of contextual knowledge in global mental health programs.

## Conclusion

We identified 38 papers, book chapters and monographs that explicitly addressed cultural dimensions of mental health and illness in Nepal. As documentation of four decades of work focused on translating among divergent approaches to understanding and addressing mental suffering, this review speaks to ongoing debates about the significance of cultural variation in psychiatric distress and disorder. Although still modest, the available literature on Nepal does not support claims that service development initiatives have completely overlooked local cultures. On the contrary, applied work done by a new generation of clinician–researchers with interdisciplinary interests and training is refining our knowledge of how culture shapes the experience, expression, and interpretation of suffering and the translatability of biomedical diagnostic categories and treatments. While this review does not speak to how well available knowledge has been applied at the level of health systems or service delivery, it suggests there is a growing interest in culturally informed mental health research and practice in Nepal. At the same time, findings suggest that applied research geared toward mental health service development still needs to engage with long-term, ethnographic studies that attend holistically to local knowledge and experience. Gaps remain in our understanding of indigenous illness concepts and healing approaches. There is a continued need to build capacity in Nepal for research driven by the needs and concerns of local stakeholders, particularly people with mental health problems and traditional healers, and to engage with open-ended methods of inquiry that recognize diverse modes of understanding mental suffering and healing.

## References

[ref1] Abramowitz SA (2010). Trauma and humanitarian translation in Liberia: the tale of Open Mole. Culture, Medicine and Psychiatry 34, 353–379. 10.1007/s11013-010-9172-0 20401629

[ref2] Acland S (2002). Mental health services in primary care: the case of Nepal In The World Mental Health Casebook: Social and Mental Health Programs in Low-Income Countries (ed. A. Cohen, A. Kleinman, B. Saraceno), pp. 121–152. Kluwer Academic/Plenum Press: New York.

[ref3] Adhikari RP, Upadhaya N, Gurung D, Luitel NP, Burkey MD, Kohrt BA, Jordans MJD (2015). Perceived behavioral problems of school aged children in rural Nepal: a qualitative study. Child and Adolescent Psychiatry and Mental Health 9, 1–9. 26131019 10.1186/s13034-015-0061-8 PMC4485359

[ref4] Alarcón RD, Becker AE, Lewis-Fernández R, Like RC, Desai P, Foulks E, Gonzales J, Hansen H, Kopelowicz A, Lu FG, Oquendo MA, Primm A (2009). Issues for DSM-V: the role of culture in psychiatric diagnosis. The Journal of Nervous and Mental Disease 197, 559–660. 19684490 10.1097/NMD.0b013e3181b0cbff

[ref5] American Psychiatric Association (2013). Diagnostic and Statistical Manual of Mental Disorders, 5th edn American Psychiatric Publishing: Washington, DC.

[ref6] Argenti-Pillen A (2003). Masking Terror: How Women Contain Violence in Southern Sri Lanka. University of Pennsylvania Press: Philadelphia.

[ref7] Arksey H, O'Malley L (2005). Scoping studies: towards a methodological framework. International Journal of Social Research Methodology 8, 19–32.

[ref8] Bhugra D, Tasman A, Pathare S, Priebe S, Smith S, Torous J, Arbuckle MR, Langford A, Alarcón RD, Chiu HFK, First MB, Kay J, Sunkel C, Thapar A, Udomratn P, Baingana FK, Kestel D, Ng RMK, Patel A, De Picker L, McKenzie KJ, Moussaoui D, Muijen M, Bartlett P, Davison S, Exworthy T, Loza N, Rose D, Torales J, Brown M, Christensen H, Firth J, Keshavan M, Li A, Onnela JP, Wykes T, Elkholy H, Kalra G, Lovett KF, Travis MJ, Ventriglio A (2017). The WPA-Lancet Psychiatry Commission on the future of psychiatry. The Lancet Psychiatry 4, 775–818. 28946952 10.1016/S2215-0366(17)30333-4

[ref9] Boehnke K, Stromberg C, Regmi MP, Richmond BO, Chandra S (1998). Reflecting the world ‘out there’: a cross-cultural perspective on worries, values and well-being. Journal of Social and Clinical Psychology 17, 227–247.

[ref10] Böker H (1992). Concepts of mental illness: an ethnopsychiatric study of the mental hospital's in- and out-patients in the Kathmandu Valley. Contributions to Nepalese Studies 19, 27–50.

[ref11] Bracken P, Giller J, Summerfield D (2016). Primum non nocere. The case for a critical approach to global mental health. Epidemiology and Psychiatric Sciences 25, 506–510. 27522868 10.1017/S2045796016000494 PMC7137661

[ref12] Bragin M, Onta K, Nzeyimana G (2014). To be well at heart: women's perceptions of psychosocial well- being in 3 conflict-affected countries – Burundi, Nepal, and Uganda. Intervention 12, 187–209.

[ref13] Burkey MD, Ghimire L, Adhikari RP, Kohrt BA, Jordans MJD, Haroz EE, Wissow LS (2016 *a*). Development process of an assessment tool for disruptive behavior problems in cross-cultural settings: the Disruptive Behavior International Scale – Nepal version (DBIS-N). International Journal of Culture and Mental Health 9, 387–398. 28093575 10.1080/17542863.2016.1226372 PMC5234690

[ref14] Burkey MD, Ghimire L, Adhikari RP, Wissow LS, Jordans MJD, Kohrt BA (2016 *b*). The ecocultural context and child behavior problems: a qualitative analysis in rural Nepal. Social Science & Medicine 159, 73–82. 27173743 10.1016/j.socscimed.2016.04.020 PMC5201200

[ref15] Campbell C, Burgess R (2012). The role of communities in advancing the goals of the Movement for Global Mental Health. Transcultural Psychiatry 49, 379–395. 23008350 10.1177/1363461512454643

[ref16] Center for Victims of Torture Nepal (2011). Community Mental Health Promotion Program in Nepal. Kathmandu.

[ref17] Chase LE, Bhattarai D (2013). Making Peace In The Heart-Mind: towards an ethnopsychology of resilience among Bhutanese refugees. European Bulletin of Himalayan Research 43, 144–166.

[ref18] Chase LE, Marahatta K, Sidgel K, Shrestha S, Gautam K, Luitel N, Dotel BR, Samuel R (2018). Building back better? Taking stock of the post-earthquake mental health and psychosocial response in Nepal. International Journal of Mental Health Systems 12, 1–12. 30083225 10.1186/s13033-018-0221-3 PMC6071401

[ref19] Chase L, Sapkota RP (2017). ‘In our community, a friend is a psychologist’: an ethnographic study of informal care in two Bhutanese refugee communities. Transcultural Psychiatry 54, 400–422. 28475482 10.1177/1363461517703023

[ref20] Chase LE, Welton-Mitchell C, Bhattarai S (2013). ‘Solving tension:’ coping among Bhutanese refugees in Nepal. International Journal of Migration, Health and Social Care 9, 71–83.

[ref21] Chen P, Ganesan S, Mckenna M (2013). Overview of psychiatric scales used in Nepal: their reliability, validity and cultural appropriateness. Asia-Pacific Psychiatry 5, 113–118. 23857938 10.1111/j.1758-5872.2012.00212.x

[ref22] Chisholm D, Flisher A, Lund C, Patel V, Saxena S, Thornicroft G, Tomlinson M (2007). Scale up services for mental disorders: a call for action. The Lancet 370, 1241–1252. 10.1016/S0140-6736(07)61242-2 17804059

[ref23] Clark J (2014). Medicalization of global health 2: the medicalization of global mental health. Global Health Action 71, 24000, DOI: 10.3402/gha.v7.24000 Available from: 10.3402/gha.v7.24000. PMC4028926 24848660

[ref24] Clarke K, Saville N, Bhandari B, Giri K, Ghising M, Jha M, Jha S, Magar J, Roy R, Shrestha B, Thakur B, Tiwari R, Costello A, Manandhar D, King M, Osrin D, Prost A (2014). Understanding psychological distress among mothers in rural Nepal: a qualitative grounded theory exploration. BMC Psychiatry 14, 1–13. Available from: http://www.biomedcentral.com/1471-244X/14/60. 10.1186/1471-244X-14-60 PMC3943437 24581309

[ref25] Cole PM, Bruschi CJ, Tamang BL (2002). Cultural differences in children's emotional reactions to difficult situations. Child Development 73, 983–996. 12038564 10.1111/1467-8624.00451

[ref26] Cole PM, Tamang BL (1998). Nepali children's ideas about emotional displays in hypothetical challenges. Developmental Psychology 34, 640–646. 9681255 10.1037//0012-1649.34.4.640

[ref27] Collins PY, Patel V, Joestl SS, March D, Insel TR, Daar AS (2011). Grand challenges in global mental health. Nature 475, 27–30. 21734685 10.1038/475027a PMC3173804

[ref28] Desjarlais RR (1989). Healing through images: the magical flight and healing geography of Nepali shamans. Ethos (Berkeley, California ) 17, 289–307.

[ref29] Desjarlais RR (1992). Body and Emotion: The Aesthetics of Illness and Healing in the Nepal Himalayas. University of Pennsylvania Press: Philadelphia.

[ref30] Desjarlais R, Eisenberg L, Good B, Kleinman A (1995). World Mental Health: Problems and Priorities in Low-Income Countries. Oxford University Press: New York.

[ref31] Dietrich A (1998). Tantric Healing in the Kathmandu Valley: A Comparative Study of Hindu and Buddhist Spiritual Healing Traditions in Urban Nepalese Society. Book Faith India: Delhi.

[ref32] Evers S, Van Der Brug M, Van Wesel F, Krabbendam L, Amsterdam VU, Studies E, Amsterdam VU (2016). Mending the levee: how supernaturally anchored conceptions of the person impact on trauma perception and healing among children (cases from Madagascar and Nepal). Children and Society 30, 423–433.

[ref33] Fernando S (2014). Globalization of psychiatry – a barrier to mental health development. International Review of Psychiatry 26, 551–557. 25343630 10.3109/09540261.2014.920305

[ref34] Furr LA (2004). Medicalization in Nepal: a study of the influence of westernization on defining deviant and illness behavior in a developing country. International Journal of Comparative Sociology 45, 131–142.

[ref35] Furr LA (2005). On the relationship between cultural values and preferences and affective health in Nepal. International Journal of Social Psychiatry 51, 71–82. 15864977 10.1177/0020764005053283

[ref36] Government of Nepal (2014). Multisectoral Action Plan for the Prevention of Non Communicable Diseases (2014–2020). Government of Nepal: Kathmandu.

[ref37] Greene MC, Jordans MJD, Kohrt BA, Ventevogel P, Kirmayer LJ, Hassan G, Chiumento A, van Ommeren M, Tol WA (2017). Addressing culture and context in humanitarian response: preparing desk reviews to inform mental health and psychosocial support. Conflict and Health 11, 1–10. DOI: 10.1186/s13031-017-0123-z. 29163666 PMC5686886

[ref38] Hanlon C, Luitel NP, Kathree T, Murhar V, Shrivasta S, Medhin G, Ssebunnya J, Fekadu A, Shidhaye R, Petersen I, Jordans M, Kigozi F, Thornicroft G, Patel V, Tomlinson M, Lund C, Breuer E, De Silva M, Prince M (2014). Challenges and opportunities for implementing integrated mental health care: a district level situation analysis from five low- and middle-income countries. PLoS ONE 9, e88437. 24558389 10.1371/journal.pone.0088437 PMC3928234

[ref39] Haroz EE, Jordans M, de Jong J, Gross A, Bass J, Tol W (2017). Measuring hope among children affected by armed conflict: cross-cultural construct validity of the children's hope scale. Assessment 24, 528–539. 26508802 10.1177/1073191115612924 PMC5835958

[ref40] Harper I (2014). Dealing with ‘multiple physical complaints’: introducing psychiatric services In Development and Public Health in the Himalaya: Reflections on Healing in Contemporary Nepal, pp. 83–102. Routledge: New York.

[ref108] Heys M, Alexander A, Medeiros E, Tumbahangphe KM, Gibbons F, Shrestha R, Manandhar M, Wickenden M, Shrestha M, Costello A, Manandhar D, Pellicano E (2017). Understanding parents' and professionals' knowledge and awareness of autism in Nepal. Autism 21, 436–449. 27197696 10.1177/1362361316646558

[ref41] Hitchcock JT (1967). A Nepalese shamanism and the classic inner Asian tradition. History of Religions 7, 149–158.

[ref42] Hitchcock JT, Jones RL (eds) (1976). Spirit Possession in the Nepal Himalayas. Aris & Phillips Ltd.: Warminster.

[ref43] Hoge EA, Tamrakar SM, Christian KM, Mahara N, Nepal MK, Pollack MH, Simon NM (2006). Cross-cultural differences in somatic presentation in patients with generalized anxiety disorder. Journal of Nervous & Mental Disease 194, 962–966. 17164637 10.1097/01.nmd.0000243813.59385.75

[ref109] Hruschka DJ, Hadley C. 2008 A glossary of culture in epidemiology. Journal of Epidemiology and Community Health 62, 947–951. 18854496 10.1136/jech.2008.076729

[ref44] Inter-Agency Standing Committee (2007). IASC Guidelines on Mental Health and Psychosocial Support in Emergency Settings. IASC: Geneva. 10.1080/09540261.2022.2147420 36502397

[ref45] Inter-Agency Standing Committee (IASC) Reference Group for Mental Health and Psychosocial Support in Emergency Settings (2015). Nepal Earthquakes 2015: Desk Review of Existing Information with Relevance to Mental Health & Psychosocial Support. Kathmandu, Nepal.

[ref46] Jack DC, Pokharel B, Subba U (2010). ‘I don't express my feelings to anyone’: how self-silencing relates to gender and depression in Nepal In Silencing the Self Across Cultures: Depression and Gender in the Social World (ed. DC Jack, A Ali), pp. 147–173. Oxford University Press: Oxford.

[ref47] Jolly A (1999). Indigenous mental health care among Gurkha soldiers based in the United Kingdom. Journal of the Royal Army Medical Corps 145, 15–17. 10216841 10.1136/jramc-145-01-04

[ref48] Jordans MJ, Keen AS, Pradhan H, Tol WA (2007). Psychosocial counselling in Nepal: perspectives of counsellors and beneficiaries. International Journal for the Advancement of Counselling 29, 57–68.

[ref49] Jordans MJD, Luitel NP, Pokhrel P, Patel V (2016). Development and pilot testing of a mental healthcare plan in Nepal. British Journal of Psychiatry 208, s21–s28. 26447173 10.1192/bjp.bp.114.153718 PMC4698553

[ref50] Jordans MJD, Sharma B (2004). Integration of psychosocial counselling in care systems in Nepal. Intervention 2, 171–180.

[ref51] Jordans MJD, Tol WA, Sharma B, van Ommeren M (2003). Training psychosocial counselling in Nepal: content review of a specialized training program. Intervention 1, 18–35.

[ref52] Kaplan AS (1999). An Exploration of the Meaning of Psychiatric Symptomatology in Rural Nepal. Unpublished doctoral dissertation University of Hawaii: Honolulu, US.

[ref53] Khenti A, Fréel S, Trainor R, Mohamoud S, Diaz P, Suh E, Bobbili SJ, Sapag JC (2016). Developing a holistic policy and intervention framework for global mental health. Health Policy and Planning 31, 37–45. 25832419 10.1093/heapol/czv016

[ref54] Kim J, Tol WA, Shrestha A, Kafle HM, Rayamajhi R, Luitel NP, Thapa L, Surkan PJ (2017). Persistent complex bereavement disorder and culture: early and prolonged grief in Nepali widows. Psychiatry: Interpersonal and Biological Processes 80, 1–16. 10.1080/00332747.2016.1213560 28409713

[ref55] Kirmayer LJ (2013). 50 years of transcultural psychiatry. Transcultural Psychiatry 50, 3–5. 23589479 10.1177/1363461513484402

[ref56] Kirmayer LJ, Pedersen D (2014). Toward a new architecture for global mental health. Transcultural Psychiatry 51, 759–776. 25358524 10.1177/1363461514557202

[ref57] Kohrt BA (2009). Vulnerable social groups in postconflict settings: a mixed methods policy analysis and epidemiology study of caste and psychological morbidity in Nepal. Intervention 7, 239–264.

[ref58] Kohrt BA (2015). The role of traditional rituals for reintegration and psychosocial well-being of child soldiers in Nepal In Genocide and Mass Violence (ed. DE Hinton, AL Hinton), pp. 369–387. Cambridge University Press: New York.

[ref59] Kohrt BA, Bourey C (2016). Culture and comorbidity: intimate partner violence as a common risk factor for maternal mental illness and reproductive health problems among former child soldiers in Nepal. Medical Anthropology Quarterly 30, 515–535. 27558762 10.1111/maq.12336 PMC5177473

[ref60] Kohrt BA, Harper I (2008). Navigating diagnoses: understanding mind-body relations, mental health, and stigma in Nepal. Culture, Medicine and Psychiatry 32, 462–491. 10.1007/s11013-008-9110-6 PMC3869091 18784989

[ref61] Kohrt BA, Hruschka DJ (2010). Nepali concepts of psychological trauma: the role of idioms of distress, ethnopsychology and ethnophysiology in alleviating suffering and preventing stigma. Culture, Medicine and Psychiatry 34, 322–352. 10.1007/s11013-010-9170-2 PMC3819627 20309724

[ref62] Kohrt BS, Jordans MJ, Tol WA, Luitel NP, Maharjan SM, Upadhaya N (2011). Validation of cross-cultural child mental health and psychosocial research instruments: adapting the Depression Self-Rating Scale and Child PTSD Symptom Scale in Nepal. BMC Psychiatry 11, 1–17. 21816045 10.1186/1471-244X-11-127 PMC3162495

[ref63] Kohrt BA, Kunz RD, Baldwin JL, Koirala NR, Sharma VD, Nepal MK (2005). ‘Somatization’ and ‘comorbidity’: a study of Jhum-Jhum and depression in rural Nepal. Ethos (Berkeley, California ) 33, 125–147.

[ref64] Kohrt BA, Kunz RD, Koirala NR, Sharma VD (2003). Validation of the Nepali version of beck anxiety inventory. Journal of Institute of Medicine 25, 1–4.

[ref65] Kohrt BA, Kunz RD, Koirala NR, Sharma VD, Nepal MK (2002). Validation of a Nepali version of the Beck Depression Inventory. Nepalese Journal of Psychiatry 2, 123–130.

[ref66] Kohrt BA, Luitel NP, Acharya P, Jordans MJD (2016). Detection of depression in low resource settings: validation of the Patient Health Questionnaire (PHQ-9) and cultural concepts of distress in Nepal. BMC Psychiatry 16, 1–14. 26951403 10.1186/s12888-016-0768-y PMC4782581

[ref67] Kohrt BA, Maharjan SM (2009). When a child is no longer a child: Nepali ethnopsychology of child development and violence. Studies in Nepali History and Society 14, 107–142. PMC6857446 31736613

[ref68] Kohrt BA, Maharjan SM, Timsina D, Griffith J (2012). Applying Nepali ethnopsychology to psychotherapy for the treatment of mental illness and prevention of suicide among Bhutanese refugees. Annals of Anthropological Practice 36, 88–112.

[ref70] Kohrt BA, Ramaiya MK, Rai S, Bhardwaj A, Jordans MJD (2015). Development of a scoring system for non-specialist ratings of clinical competence in global mental health: a qualitative process evaluation of the Enhancing Assessment of Common Therapeutic Factors (ENACT) scale. Global Mental Health 2, e23. 28593049 10.1017/gmh.2015.21 PMC5269630

[ref71] Kohrt BA, Speckman RA, Kunz RD, Baldwin JL, Upadhaya N, Acharya NR, Sharma VD, Nepal MK, Worthman CM (2009). Culture in psychiatric epidemiology: using ethnography and multiple mediator models to assess the relationship of caste with depression and anxiety in Nepal. Annals of Human Biology 36, 261–280. 19381985 10.1080/03014460902839194 PMC3907946

[ref72] Kohrt BA, Upadhaya N, Luitel NP, Maharjan SM, Kaiser BN, Macfarlane EK, Khan N (2014). Authorship in global mental health research: recommendations for collaborative approaches to writing and publishing. Annals of Global Health 80, 134–142. 24976552 10.1016/j.aogh.2014.04.007 PMC4155487

[ref73] Luitel NP, Jordans MJ, Adhikari A, Upadhaya N, Hanlon C, Lund C, Komproe IH (2015). Mental health care in Nepal: current situation and challenges for development of a district mental health care plan. Conflict and Health 9, 1–11. 25694792 10.1186/s13031-014-0030-5 PMC4331482

[ref74] Maharjan SM (2012). Bibliography of Psychological Research in Nepal. Martin Chautari: Kathmandu.

[ref75] Maskarinec GG (1992). A shamanic etiology of affliction in Western Nepal. Social Science & Medicine 35, 723–734. 1439922 10.1016/0277-9536(92)90010-n

[ref76] Mchugh E (1989). Concepts of the person among the Gurungs of Nepal. American Ethnologist 16, 75–86.

[ref77] Mendenhall E, De Silva MJ, Hanlon C, Petersen I, Shidhaye R, Jordans M, Luitel N, Ssebunnya J, Fekadu A, Patel V, Tomlinson M, Lund C (2014). Acceptability and feasibility of using non-specialist health workers to deliver mental health care: stakeholder perceptions from the PRIME district sites in Ethiopia, India, Nepal, South Africa, and Uganda. Social Science & Medicine 118C, 33–42. 10.1016/j.socscimed.2014.07.057 PMC4167946 25089962

[ref78] Mills C (2014). Decolonizing Global Mental Health: The Psychiatrization of the Majority World. Routledge: New York.

[ref79] Napier AD, Ancarno C, Butler B, Calabrese J, Chater A, Chatterjee H, Guesnet F, Horne R, Jacyna S, Jadhav S, Macdonald A, Neuendorf U, Parkhurst A, Reynolds R, Scambler G, Shamdasani S, Smith SZ, Stougaard-Nielsen J, Thomson L, Tyler N, Volkmann A-M, Walker T, Watson J, de Williams ACC, Willott C, Wilson J, Woolf K (2014). Culture and health. The Lancet 384, 1607–1639. 10.1016/S0140-6736(14)61603-2 25443490

[ref80] Nichter M (1981). Idioms of distress: alternatives in the expression of psychosocial distress: a case study from South India. Culture, Medicine and Psychiatry 5, 379–408. 10.1007/BF00054782 7326955

[ref81] Pach A III (1998). Narrative constructions of madness in a Hindu village in Nepal In Selves in Time and Place: Identities, Experience, and History in Nepal (ed. D Skinner, A Pach III, D Holland), pp. 111–128. Rowman & Littlefield Publishers, Inc.: Lanham.

[ref82] Patel V, Chisholm D, Parikh R, Charlson FJ, Degenhardt L, Dua T, Ferrari AJ, Hyman S, Laxminarayan R, Levin C, Lund C, Medina Mora ME, Petersen I, Scott J, Shidhaye R, Vijayakumar L, Thornicroft G, Whiteford H (2016). Addressing the burden of mental, neurological, and substance use disorders: key messages from Disease Control Priorities, 3rd edition. *The* Lancet 387, 1672–1685. 10.1016/S0140-6736(15)00390-6 26454360

[ref83] Patel V, Collins PY, Copeland J, Kakuma R, Katontoka S, Lamichhane J, Naik S, Skeen S (2011). The movement for global mental health. British Journal of Psychiatry 198, 88–90. 21282777 10.1192/bjp.bp.109.074518 PMC3031652

[ref84] Peters L (1978). Psychotherapy in Tamang shamanism. Ethos (Berkeley, California ) 6, 63–91.

[ref85] Peters L (1981). Ecstasy and Healing in Nepal: An Ethnopsychiatric Study of Tamang Shamanism. Undena Publications: Malibu.

[ref86] Psychosocial Working Group (2003). Psychosocial Intervention in Complex Emergencies: A Conceptual Framework. Edinburgh.

[ref87] Sapkota R, Gurung D, Neupane D, Shah S, Kienzler H, Kirmayer LJ (2014). A village possessed by ‘witches’: a study of possession and common mental disorders in Nepal. Culture, Medicine and Psychiatry 38, 642–668. 10.1007/s11013-014-9393-8 25234302

[ref88] Seale-Feldman A, Upadhaya N (2015). *Mental Health after the Earthquake: Building Nepal's Mental Health System in Times of Emergency* Hotspots, Cultural Anthropology Website, October 14 2015. Available from: https://culanth.org/fieldsights/736-mental-health-after-the-earthquake-building-nepal-s-mental-health-system-in-times-of-emergency.

[ref89] Sharma B, van Ommeren M (1998). Preventing torture and rehabilitating survivors in Nepal. Transcultural Psychiatry 35, 85–97.

[ref90] Sherchan S, Samuel R, Marahatta K, Anwar N, Van Ommeren MH, Ofrin R (2017). Post-disaster mental health and psychosocial support: experience from the 2015 Nepal earthquake. WHO South-East Asia Journal of Public Health 6, 22–29. 10.4103/2224-3151.206160 28597855

[ref91] Skultans V (1988). A comparative study of the psychiatric practice of a tantrik healer and a hospital out-patient clinic in the Kathmandu Valley. Psychological Medicine 18, 969–981. 3270839 10.1017/s0033291700009892

[ref92] Sochos A, Lokshum C (2017). Adolescent attachment in Nepal: testing the factorial validity of two scales. European Journal of Developmental Psychology 14, 498–508.

[ref93] Soubrouillard B (1995). A Psychological Study of the Vision and Treatment of Mental Illness by Nepalese Shamans. Unpublished doctoral dissertation Pacifica Graduate Institute: Carpinteria, USA.

[ref94] Stone L (1976). Concepts of illness and curing in a central Nepal village. Contributions to Nepalese Studies 3, 54–82.

[ref95] Subba S (2007). *Socio-Cultural Construction of Illness: Oral Recitals of Genesis, Causes and Cure of Roga (naturally caused illnesses)*. Ms. Usha Kiran Subba: Kathmandu, Nepal Available from: https://www.researchgate.net/profile/Shishir_Subba/publication/220024800_Sociocultural_Construction_of_Illness_Oral_Recitals_of_Genesis_Causes_and_Cure_of_Naturally_Caused_Illnesses/links/00463515716d542b08000000/Sociocultural-Construction-of-Illness-Oral-Recitals-of-Genesis-Causes-and-Cure-of-Naturally-Caused-Illnesses.pdf

[ref96] Subba P, Luitel NP, Kohrt BA, Jordans MJD (2017). Improving detection of mental health problems in community settings in Nepal: development and pilot testing of the community informant detection tool. Conflict and Health 11, 28. 29181088 10.1186/s13031-017-0132-y PMC5694900

[ref97] Summerfield D (2012). Afterword: against ‘global mental health’. Transcultural Psychiatry 49, 519–530. 23008353 10.1177/1363461512454701

[ref98] Tol WA, Jordans MJD, Regmi S, Sharma B (2005). Cultural challenges to psychosocial counselling in Nepal. Transcultural Psychiatry 42, 317–333. 16114588 10.1177/1363461505052670

[ref99] Tol WA, Kohrt BA, Jordans MJD, Thapa SB, Pettigrew J, Upadhaya N, de Jong JTVM (2010). Political violence and mental health: a multi-disciplinary review of the literature on Nepal. Social Science & Medicine (1982) 70, 35–44. 19833427 10.1016/j.socscimed.2009.09.037

[ref100] United Nations Development Programme (2016). Human Development Report 2016. United Nation Development Programme, New York.

[ref101] Upadhaya N, Luitel NP, Koirala S, Ramesh P, Gurung D, Shrestha P, Tol WA, Brandon A, Jordans MJD (2014). The role of mental health and psychosocial support nongovernmental organisations: reflections from post conflict Nepal. Intervention 12, 113–128.

[ref102] van Ommeren M, Sharma B, Thapa S, Makaju R, Prasain D, Bhattarai R, de Jong J (1999). Preparing instruments for transcultural research: use of the translation monitoring form with Nepali-speaking Bhutanese refugees. Transcultural Psychiatry 36, 285–301.

[ref103] Watters E (2010). Crazy Like Us: The Globalization of the Western Mind. Robinson: London.

[ref104] Whitley R (2015). Global Mental Health: concepts, conflicts and controversies. Epidemiology and Psychiatric Sciences 24, 285–291. 26027857 10.1017/S2045796015000451 PMC7192180

[ref105] World Health Organization (2011). Mental Health Atlas 2011: Nepal. Geneva.

[ref106] World Health Organization (2013 *a*). Building Back Better: Sustainable Mental Health Care after Emergencies. Geneva.

[ref107] World Health Organization (2013 *b*). Mental Health Action Plan 2013–2020. Geneva.

